# Resource acquisition and pre‐copulatory sexual selection

**DOI:** 10.1002/ece3.9137

**Published:** 2022-07-24

**Authors:** Hope Klug, Chelsea Langley, Elijah Reyes

**Affiliations:** ^1^ Department of Biology, Geology, and Environmental Science University of Tennessee at Chattanooga Chattanooga Tennessee USA; ^2^ SimCenter University of Tennessee at Chattanooga Chattanooga Tennessee USA; ^3^ Department of Biological Sciences Simon Fraser University Burnaby British Columbia Canada

**Keywords:** mate availability, mate competition, mate limitation, resource availability, resource competition, sexual selection

## Abstract

Sexual selection influences the evolution of phenotypic traits and contributes to patterns of biodiversity. In many animals, mating involves sequential steps. Often, individuals must secure resources that are essential for mating (nests, territories, food), and then after securing a resource, individuals engage in competition for access to limited opposite sex mates and gametes. A large body of empirical research and some verbal models have illustrated that resource acquisition can influence sexual selection. In general, though, we lack *a priori* predictions of when and how resource acquisition will influence sexual selection. Here, we use a mathematical framework to explore the link between resource acquisition and sexual selection on an advantageous mate‐acquisition trait across biologically relevant trade‐off scenarios. Our findings provide a set of testable predictions of how resource acquisition can influence sexual selection on mating traits. In general, selection on mate‐acquisition traits is expected to be heavily influenced by: (1) the episode of selection considered, and in particular, whether one considers selection associated with the mating pool only or selection associated with both the mating pool and pre‐mating pool; (2) whether resource‐acquisition and mate‐acquisition traits are positively associated or whether they trade off; and (3) the proportion of males with the resource‐ and mate‐acquisition traits.

## Graphical abstract

Resource competition can have cascading effects on sexual selection.

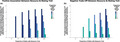

## 1 INTRODUCTION

Sexual selection (i.e., covariation between one or more traits, mating success, and the fertilization success of limited opposite sex gametes (Shuker & Kvarnemo, 2021)) influences micro‐ and macro‐evolutionary patterns of biodiversity (Dunn et al., 2015; Emlen et al., 2012; Fromhage & Jennions, 2016; Harrison et al., 2015; Janicke et al., 2016; Lumley et al., 2015; Servedio & Boughman, 2017). Sexual selection is affected by numerous factors (Kokko et al., 2012; Shuker, 2010; Shuker & Kvarnemo, 2021), including the operational sex ratio (OSR: ratio of males and females prepared to mate at a given place and time (Emlen & Oring, 1977), adult sex ratio (ASR: ratio of adult males to females (Kokko & Jennions, 2012)), reproductive rate (Ahnesjö et al., 2001; Clutton‐Brock & Parker, 1992; Clutton‐Brock & Vincent, 1991), mate quality (Johnstone, 1995), adult mortality (Kokko & Jennions, 2008; Liker & Székely, 2005; Vollrath & Parker, 1992), breeding costs (Kokko & Monaghan, 2001), population density (Klug et al., 2010; Kokko & Rankin, 2006), and parental investment (Fromhage & Jennions, 2016; Kokko & Jennions, 2008; Székely et al., 2000). In addition, resource availability can have strong effects on sexual selection in relation to mate monopolization (Emlen & Oring, 1977), mate signaling and attraction (Borgia et al., 1987; Dawkins, 1982; Enquist, 1985; Grafen, 1990; Head et al., 2017; Johnstone et al., 2009; Pärssinen et al., 2019; Penn & Számadó, 2019; Rowe & Houle, 1996; Schaedelin & Taborsky, 2009; Zahavi, 1975, 1977), and the benefits of mate choice (Andersson, 1994; Miller & Svensson, 2014). Furthermore, resource availability affects sexual selection by determining which individuals and traits enter the mating pool, where the mating pool consists of the individuals that are currently prepared to mate at a current time and location (Ahnesjö et al., 2001; Kokko et al., 2012; Shuker & Kvarnemo, 2021). Specifically, acquiring mates and limited opposite sex gametes often involves multiple, sequential steps (Ahnesjö et al., 2001; Brown et al., 2008, 2010; Klug et al., 2010; West‐Eberhard, 1983). In many cases, individuals of a given sex must compete for and acquire resources that are required for mating (e.g., nests, territories, food), and only after acquiring such resources can individuals enter the mating pool and engage in direct mate competition and/or attempt to be chosen as a mate (Ahnesjö et al., 2001). Such pre‐mating pool resource acquisition can be influenced by both natural and sexual selection (discussed in (Shuker & Kvarnemo, 2021), and both general resource and direct mate acquisition can affect sexual selection (Ahnesjö et al., 2001; Klug et al., 2010).

Empirically, the availability of nests, bowers, and territories—critical resources for mating in many species—has been found to strongly influence sexual selection. In the sand goby (*Pomatoschistus minutus*), for instance, parental males compete for and acquire nests prior to engaging in direct mate competition (Lindström, 1988, 2001; Singer et al., 2006). Forsgren et al. (1996) found that nest limitation was associated with relatively greater intra‐sexual selection, whereas an abundance of nests was associated with relatively greater inter‐sexual selection across populations. Similarly, in the common goby (*Pomatoschistus microps*), males engaged in more courtship when nests were abundant, and in contrast, females engaged in more courtship and female–female aggression, whereas males engaged in greater sneaking attempts, when nests were limited (Borg et al., 2002). In the blenniid fish *Salaria pavo*, scarce nest sites led to female courtship behavior (Almada et al., 1995), and in the two‐spotted goby (*Gobiusculus flavescens*), nest distribution influenced aggressive interactions among males (Mück et al., 2013) and is inter‐related with direct mate competition (Wacker & Amundsen, 2014). Nests and territories can also be directly involved in mate assessment. In the great reed warbler (*Acrocephalus arundinaceus*), nest size can serve as an indicator of female quality and influence male investment (Jelínek et al., 2015), and female bowerbirds show strong preferences for particular characteristics of males' bowers (Madden & Tanner, 2003), including scarce blue flowers (Borgia et al., 1987). Female common gobies also base their mating decisions on both male phenotype and nest characteristics (Pärssinen et al., 2019).

Beyond nests, territories, and bowers, food availability also influences mating rate and can impact sexual selection (Miller & Svensson, 2014). In the sand goby, the female inter‐spawning interval was smaller when food was abundant versus scarce, whereas the inter‐spawning interval of males was not influenced by food abundance (Kvarnemo, 1997). As such, relatively food‐limited conditions are expected to be associated with a male‐biased OSR and greater male–male competition (Kvarnemo, 1997). Across sand goby populations, a greater proportion of females were prepared to mate in a population in which food availability was high versus low, which could lead to greater male choosiness and female–female competition (García‐Berro et al., 2019). Similarly, in a freshwater snail (*Physa acuta*), reduced food led to lower variance in male mating success and a reduced potential for sexual selection, as well as greater post‐copulatory sexual selection in males (Janicke et al., 2015). Food availability also affects the strength of sexual selection when females receive accessory ejaculate substances from males (Arnqvist & Nilsson, 2000). In insects, nuptial feeding by males can increase female mating rate (reviewed in Arnqvist & Nilsson, 2000). When male nuptial gifts serve as an alternative food source, there is potential for greater sexual selection on female traits when food is scarce because males become a limited resource (Gwynne & Simmons, 1990). Food availability also influences sex roles in the flower‐feeding bush cricket (*Kawanaphila nartee*) (Hare & Simmons, 2020; Kvarnemo & Simmons, 1999). When food is relatively scarce, females compete for male nuptial gifts and males are choosy, whereas when food is abundant, males compete for females (Hare & Simmons, 2020). Likewise, male tettigoniids, who provide nutrients to female during mating, were choosy and females were competitive when food availability was low; when food was abundant, females exhibited mate choice and males were less discriminate (Simmons & Bailey, 1990). Feeding by males during mating also influences sexual selection in birds. In the polygamous feral fowl (*Gallus g. domesticus*), males provide female mates with food and females prefer males that provide relatively more food (Pizzari, 2003). Collectively, these empirical studies suggest that the availability of general, non‐mate resources can have strong effects on mating and sexual selection.

The importance of resource availability has been noted in conceptual studies of sexual selection. Emlen and Oring (1977) hypothesized that the strength of sexual selection will increase when there is greater potential for resource monopolization. They suggested that as resources become clumped in space and/or time, relatively few males have the potential to monopolize those resources at the exclusion of other males; because females require such resources, those males will also have greater potential to monopolize female mates when resources are clumped, which in turn is expected to increase the strength of sexual selection (Emlen & Oring, 1977). Furthermore, as noted by Ahnesjö et al. (2001), sexual selection can arise from resource competition, mate competition, and/or sperm competition. Unlike mate competition, resource competition typically cannot be predicted by sexual differences in the potential reproductive rate (PRR: the offspring production per unit time each sex would achieve if unconstrained by mate availability) or by sex differences in OSR (Ahnesjö et al., 2001). Importantly, nest and mating competition also potentially lead to selection on different traits (Ahnesjö et al., 2001). Because resource and mate competition can be distinct processes, Ahnesjö et al. (2001) proposed the concept of the Qualified Sex Ratio (Q), which is a metric that includes only those adult individuals who are currently prepared to mate and have acquired the prerequisites necessary for mating (nutritional resources, nests, etc.) (Ahnesjö et al., 2001). Ahnesjö et al. (2001) suggest that Q will, in some cases, predict the intensity of mate competition and sexual selection, because unlike ASR, Q does not confound resource and mating competition.

Together, the above research suggests that general, non‐mate resource availability can influence the abundance and distribution of individuals and traits in the mating pool, which can in turn influence mate competition, mate choice, and sexual selection on traits. Such resource competition can be influenced by natural and/or sexual selection (discussed in (Shuker & Kvarnemo, 2021)). Because resource acquisition influences mating pool dynamics, a complete understanding of the operation of sexual selection necessitates an understanding of how and when resource acquisition will affect sexual selection regardless of whether resource acquisition is influenced by natural and/or sexual selection. That is, regardless of whether resource acquisition is shaped by natural or sexual selection, resource acquisition is expected to influence selection on traits associated with mate acquisition, and hence, it is critical to understand how resource acquisition can shape the selection on traits associated with mating and the fertilization of limited opposite sex gametes. While there is substantial empirical evidence suggesting that non‐mate resource availability influences sexual selection (e.g., Forsgren et al., 1996; Ghislandi et al., 2018; Hasegawa, 2018; Hasegawa et al., 2012; Tudor et al., 2018; Vitousek, 2009; Wacker & Amundsen, 2014; Wong et al., 2018), and ongoing calls for a deeper understanding of the effects of intra‐sexual resource availability on sexual selection (Ahnesjö et al., 2001; Clutton‐Brock, 2009; Clutton‐Brock et al., 2006; Klug et al., 2010; Lyon & Montgomerie, 2012; Shuster & Wade, 2003), there is relatively little formal theory that explicitly explores the effect of resource acquisition on sexual selection (Clutton‐Brock & Parker, 1992; Clutton‐Brock & Vincent, 1991; Kokko & Jennions, 2008). As such, in general, we lack *a priori* hypotheses of how and when sexual selection will be influenced by resource acquisition. Here, we begin to bridge this gap by using a mathematical framework to explore the effect of resource acquisition on sexual selection across basic biological trade‐off scenarios. In doing so, our aim is to generate a set of *a priori* predictions of the effect of resource acquisition on sexual selection.

## 2 METHODS

### 2.1 Model overview

**FIGURE 1:**
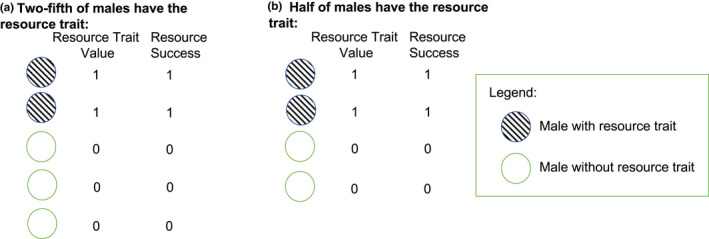
An illustration of resource trait abundance and resource success in the model. We consider scenarios in which males must acquire a discrete resource (e.g., nest, territory, bower) to enter the mating pool. In the model, males either have or lack a trait that allows them to acquire a single resource. The resource trait value is equal to one if males have the resource trait and zero if males lack the resource trait. (a) if we imagine a scenario in which two‐fifths of adult males in the population have a resource trait, only those two males who acquire the resource would be successful at resource acquisition and enter the mating pool. (b) if we imagine a scenario in which two‐fourths of adult males in the population have a resource trait, only those two males would be successful at resource acquisition and enter the mating pool.

**FIGURE 2:**
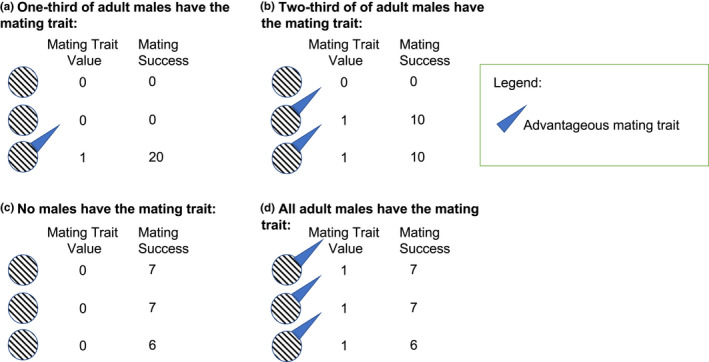
An illustration of mating trait abundance and mating success in the model. We consider scenarios in which males must acquire a discrete resource to enter the mating pool. In the current figure, we focus on males that have acquired the resource and entered the mating pool. Once in the mating pool, males are assumed to either possess or lack a trait that is advantageous in mate acquisition. In the model, males either have or lack the advantageous mating trait. The mating trait value is equal to one if males have the mating trait and zero if males lack the mating trait. Because females preferentially mate with males that have the mating trait, female mates are assigned as equitably as possible among males that possess the mating trait while maintaining an integer value for mating success. Let us imagine that there are 20 sexually receptive females in the populations. (a) If we imagine a scenario in which one‐third of adult males have the mating trait, all 20 females will mate with the male with the mating trait. (b) If two‐thirds of adult males have the mating trait, only those two males will be successful at mate acquisition and each male will mate with 10 female mates. (c) If no males in the mating pool possess the mating trait, female mates will be equitably distributed among all males present in the mating pool while maintaining an integer value for mating success. (d) If all males were to possess the mating trait, female mates will be equitably distributed among all males present in the mating pool while maintaining an integer value for mating success.

We use toy models (i.e., deliberately simple models (Otto & Day, 2007)) to illustrate the effect that resource acquisition can have on sexual selection. We intentionally focus on relatively simple and intuitive scenarios to provide a set of baseline hypotheses of how resource acquisition can influence sexual selection. In the model, males either have or do not have (1) a trait that allows them to acquire a resource that is essential for mating (resource trait) and (2) a trait that allows them to directly acquire one or more mates once in the mating pool (mating trait). Males must acquire a discrete resource (e.g., nest, territory, bower) before they can enter the mating pool and attempt to acquire a mate. In the model, each male can acquire only one resource, and as such the resource trait could, for example, be thought of as a trait that allows males to acquire a territory, nest, bower, or other discrete resource that is necessary for reproduction (Figure 1). The resource trait could be a trait that gives males an advantage in resource competition or a trait that simply allows a male to locate and maintain a resource. Such a trait could be favored by natural and/or sexual selection (discussed below; see also (Shuker & Kvarnemo, 2021)). After acquiring a resource, a given male enters the mating pool and can attempt to acquire one or more female mates. Whether a male who is in the mating pool will acquire female mate(s) will depend on whether he has the mating trait, and females are assumed to mate with males with the mating trait over those who lack this trait (Figure 2). The mating trait can therefore be thought of as any discrete trait that is preferred during mate choice, a trait that gives males an advantage during direct mate competition, or a trait that simply allows males to locate females more efficiently. For simplicity, the resource and mating traits are assumed to be binary (i.e., males either have or do not have a resource and/or mating trait; Figures 1, 2). In the model, we assume that males can mate multiply, whereas females can mate only once during a given reproductive episode. As such, mate availability only affects male fitness, and we focus on selection on male traits. Using this basic modeling framework, we consider varying levels of male resource trait abundance and male mating trait abundance across biologically relevant trade‐off scenarios (described below; Table 1). Across these scenarios, we quantify the strength of selection on the resource and mating traits, and this allows us to explore (1) how resource and mate acquisition can independently and interactively affect selection associated with resource acquisition and mating, and (2) whether selection associated with resource acquisition can have cascading effects that subsequently affect sexual selection on a mating trait.

**TABLE 1:** Overview of modeling scenarios. In the model, we considered two general relationships between the resource and mating traits. We then considered six levels of resource trait abundance crossed with six levels of mating trait abundance.

Trade‐off scenario	Proportion of males with resource trait	Proportion of males with mating trait
Resource‐acquisition and mate‐acquisition traits are positively associated	0.1, 0.3, 0.5, 0.7, 0.9, or 1 adult males have the resource trait	0.1, 0.3, 0.5, 0.7, 0.9, or 1 adult males have the mating trait
Resource‐acquisition and mate‐acquisition traits are negatively associated	0.1, 0.3, 0.5, 0.7, 0.9, or 1 adult males have the resource trait	0.1, 0.3, 0.5, 0.7, 0.9, or 1 adult males have the mating trait

### 2.2 Modeling scenarios

Traits associated with resource and mate acquisition can be positively or negatively associated with one another depending on whether some individuals are simply ‘better’ than others or whether trade‐offs exist. To account for the fact that resource‐ and mate‐acquisition traits can be positively or negatively associated, we considered the following trade‐off scenarios: (1) The case in which some males are ‘better’ than others such that there is a positive association between having the resource and mating traits; and (2) the case in which there is a trade‐off between the resource and mating traits (e.g., because males have limited energy to invest in such traits), such that there is a negative association between the resource and mating traits. For both trade‐off scenarios, we crossed six levels of male resource‐trait abundance with six levels of male mating‐trait abundance and explored the effect of resource‐ and mating‐trait abundance on selection on male traits (Table 1).

For each reproductive episode and each of the two trade‐off scenarios described above, we focused only on adult dynamics and considered an initial population of 20 adult males and 20 adult females. In the model, males are initially excluded from the mating pool and must acquire a resource to acquire a mate. As such, resource acquisition determines which adult males enter the mating pool, and resource acquisition can thus be thought of as a gatekeeper to mating, such that adult males who are unable to secure a resource cannot mate. We considered six levels of male resource‐trait abundance (0.1, 0.3, 0.5, 0.7, 0.9, or 1 = the proportion of adult males with the resource trait) crossed with six levels of male mating‐trait abundance (0.1, 0.3, 0.5, 0.7, 0.9, or 1 = proportion of males with mating trait) (Table 1). As mentioned above, males must have a resource to acquire a female mate, and females preferentially mate with males that have the mating trait. While females preferentially mate with males that possess the mating trait, females will mate with a male that lacks the mating trait if no males possess the mating trait. Thus, the mating trait is beneficial, but not essential, for mate acquisition in some scenarios.

### 2.3 Mating success and strength of selection calculations

For each trade‐off scenario and each combination of male resource‐trait abundance and mating‐trait abundance, we, as mentioned above, considered an initial population of 20 adult males and 20 adult females. Resources were then assigned among males in relation to their resource‐trait value, as illustrated in Figure 1. The mating trait was assigned to each male based on the mating‐trait abundance considered (0.1, 0.3, 0.5, 0.7, 0.9, or 1 = proportion of males with the mating trait) and the trade‐off scenario assumed. For cases in which a positive association existed between the resource and mating traits, there was a direct and positive association between a male possessing both the resource and mating traits, such that males with the resource trait were more likely to possess the mating trait while still maintaining the trait abundances for a given scenario; for cases in which a negative association existed between the resource and mating traits, there was a direct and negative relationship between whether a male had both the resource and mating traits, such that males without the resource trait were more likely to possess the mating trait while still maintaining the trait abundances for a given scenario. Given that the resource and mating traits are discrete, when there is (1) a positive association between the two traits and (2) the proportion of males with the resource trait equals the proportion of males with the mating trait, the males with the resource trait will necessarily possess the mating trait. When the proportion of males with the resource trait differs from that of the mating trait, males who have the resource trait will also have the mating trait (and vice versa) to whatever extent possible while still maintaining a given level of resource‐ and mating‐trait abundances. For example, when there are more males with the resource trait than the mating trait, all males with the mating trait will have the resource trait, but a number of males (i.e., the difference between males with the mating trait and those with the resource trait) will only have the resource trait. Likewise, when there is a negative association between the two traits, the males who have the resource trait will be different from the males that have the mating trait (and vice versa) to whatever extent possible while still maintaining a given level of resource‐ and mating‐trait abundances. If the sum of the proportions of males with the mating trait and the resource trait is greater than one, a number of males will have both traits despite the traits being negatively associated. For example, if the resource‐ and mating‐trait proportions are each 0.5 or less, no males with the resource trait will also have the mating trait. However, when the resource‐ and mating‐trait proportions are both >0.5, a number of males who possess one trait will have both traits.

Female mates were equitably assigned among the males who had both traits (i.e., all males with both traits had an equal likelihood of acquiring each female mate) (Figure 2), and in the case in which no males had both the resource and mating trait, female mates were equitably assigned among the males who had the essential resource trait and were therefore in the mating pool (Figures 1, 2). In all cases, female mates were assigned as equitably as possible to males with the advantageous traits while maintaining an integer value for mating success (Figure 2). This is essential for biological realism since it is impossible to acquire a fraction of a mate (discussed in Klug & Stone, 2021). We assumed that mating success was directly proportional to fertilization success of limited opposite sex gametes. Thus, all sexual selection in our model stems from variation in mate acquisition success.

To explore the effect of resource acquisition on mating dynamics, we calculated the strength of selection associated with the possible episodes of selection in our model. In all cases the strength of selection was quantified as the selection differential or sexual selection differential, which quantifies the strength of selection on the phenotypic trait and is proportional to the response to selection assuming heritable variation associated with the trait (Jones, 2009). The selection differentials were calculated as the covariance between the trait value (mating or resource trait = 0 or 1) and relative resource or relative mating success (Jones, 2009). All analyses were also performed using standardized selection differentials, and the qualitative patterns were identical to the results using selection differentials.

To explore the selection associated directly with pre‐mating pool resource acquisition, we first calculated the selection differential associated with the resource trait (*s*_resource_) as follows:

(1) $$s_{\text{resource}}=\mathrm{cov}\left(x_{\text{resource}},W_{\text{resource}}\right)$$

where *x*_resource_ is the male resource‐trait value, *W*_resource_ is relative resource success. All adult males were included in this selection calculation since all adult males either have or lack the resource trait.

To explore directly the selection associated with only mating pool dynamics (i.e., the selection associated with direct mate acquisition), we then calculated the sexual selection differential associated with the mating trait (*s*_mating_matpool_) as follows:

(2) $$s_{\text{mating}\_\text{matpool}}=\mathrm{cov}\left(x_{\text{mating}},W_{\text{mating}}\right)$$

where *x*_mating_ is the male mating trait value, and *W*_mating_ is relative mating success (Jones, 2009). Only males who acquired a resource and were therefore in the mating pool were included in this calculation of the selection differential on the mating trait. That is, males who lacked a resource and were therefore unable to acquire mates were excluded from this calculation of sexual selection strength, which allowed us to explore the sexual selection associated with direct mate acquisition within the mating pool.

To explore the overall or combined effect of both resource‐trait abundance and mating‐trait abundance on selection on the mating trait, we also calculated a combined measure of selection on the mating trait that accounts for both pre‐mating pool and mating pool dynamics. In this case, we calculated the overall selection differential associated with the mating trait by including all adult males in our analyses (i.e., both adult males who acquired a resource and those who lacked a resource). This measure, *s*_mating_allmales_, was calculated as follows:

(3) $$s_{\text{mating}\_\text{allmales}}=\mathrm{cov}\left(x_{\text{mating}},W_{\text{mating}}\right)$$

This measure of selection allowed us to collectively explore the effect of resource‐trait abundance and mating‐trait abundance on the overall strength of selection on the mating trait across our trade‐off scenarios. Because resource acquisition could be influenced by natural and/or sexual selection, *s*_mating_allmales_ potentially reflects the effects of both natural and/or sexual selection on the mating trait.

Importantly, traits associated with resource acquisition can be under natural and/or sexual selection (Shuker & Kvarnemo, 2021). In particular, when resources themselves directly impact access to limited opposite sex gametes, sexual selection will favor traits that allow for the acquisition of those resources (Shuker & Kvarnemo, 2021). Thus, *s*_resource_ could be a measure of natural selection, sexual selection, or some combination of natural and sexual selection, and the relative contribution of natural and/or sexual selection will depend on the specific trait considered for a given system. Bower or territory acquisition, for example, is expected to be under only sexual selection, whereas the acquisition of nests or food resources that are necessary for mating is likely to be under natural selection or a combination of natural and sexual selection. Regardless of whether resource acquisition is under natural and/or sexual selection, the scenarios considered in our model allow us to explore the cascading impact of resource acquisition on subsequent sexual selection on an advantageous mating trait.

We hypothesized that the strength of selection on the resource and mating traits will be relatively strong when few males have resource or mating traits, respectively, as these are the conditions under which relatively few males will monopolize multiple females. In addition, because the resource trait determines who can enter the mating pool, we expected that resource‐trait abundance will, in some cases, affect the strength of sexual selection on the mating trait.

## 3 RESULTS

### 3.1 Natural and/or sexual selection associated with pre‐mating pool dynamics

**FIGURE 3:**
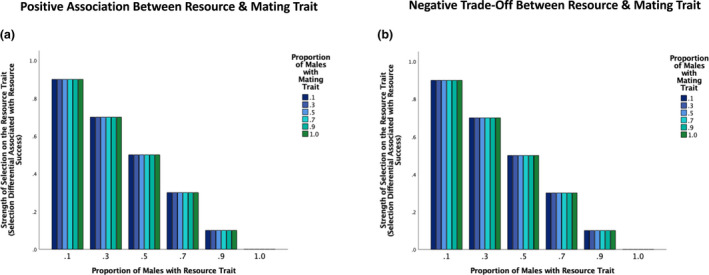
The strength of selection on a male resource‐acquisition trait. The selection differential associated with a resource trait across six levels of resource‐trait abundance and six levels of mating‐trait abundance (proportion of males with the resource trait = 0.1, 0.3, 0.5, 0.7, 0.9, or 1, proportion of males with the mating trait = 0.1, 0.3, 0.5, 0.7, 0.9, or 1) when there is (a) a positive association and (b) a negative trade‐off between the resource and mating traits.

There was greater selection on the male resource trait when a smaller proportion of males had the resource trait regardless of whether the resource and mating trait were positively associated or traded off (Figure 3a,b). This pattern was consistent across levels of male mating‐trait abundance (Figure 3a,b). This result is intuitive and expected since the resource trait is directly linked with resource acquisition in the model.

### 3.2 Sexual selection associated with mating pool dynamics

**FIGURE 4:**
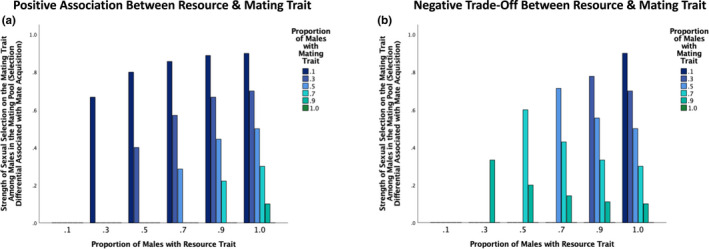
The strength of sexual selection on a male mate‐acquisition trait in relation to mating pool dynamics. The selection differential associated with a mating trait across six levels of resource‐trait abundance and six levels of mating‐trait abundance (proportion of males with the resource trait = 0.1, 0.3, 0.5, 0.7, 0.9, or 1, proportion of males with the mating trait = 0.1, 0.3, 0.5, 0.7, 0.9, or 1) when there is (a) a positive association and (b) a negative trade‐off between the resource and mating traits. To focus only on sexual selection associated with the mating pool, only males who had a resource and were therefore prepared to mate were included in the selection differential calculations.

When we quantified the selection directly associated with mate acquisition by focusing only on those males in the mating pool (i.e., those males who had a resource and were therefore currently prepared to mate), the strength of sexual selection on the mating trait was influenced by the proportion of males that had the mating trait and the proportion of males that had the resource trait (Figure 4). When there was a positive association between the resource and mating traits, the strength of sexual selection was greatest when all males had the resource trait and relatively few males (10%) had the mating trait (Figure 4a). On average, when there was a positive association between the resource and mating traits, the strength of sexual selection associated with direct mate acquisition increased as the proportion of males with the mating trait decreased and as the proportion of males with the resource trait increased (Figure 4a). In some cases, though, sexual selection in relation to mate acquisition was inhibited when the resource and mating traits were positively associated. This inhibition of sexual selection within the mating pool occurred when few males had the resource trait, particularly when many males had the mating trait (Figure 4a).

When the resource and mating traits traded off, the strength of sexual selection associated with mating pool dynamics was greatest when all males had the resource trait and few males had the mating trait (Figure 4b). This scenario is equivalent for positive and negative associations between the resource and mating traits as, under this condition, all males are in the mating pool but few have the mating trait, which leads to strong mate monopolization among mating pool males with the mating trait. When there was variation among males with respect to the resource and mating traits, the strength of sexual selection associated with mating pool dynamics tended to, on average, increase as: (1) a smaller proportion of males had the mating trait; and (2) a smaller proportion of males had the resource trait for a given level of mating‐trait abundance when sexual selection occurred with respect to mating pool dynamics (Figure 4b). However, the strength of sexual selection was highly dependent on the specific combination of resource‐ and mating‐trait abundances considered (Figure 4b). When the resource and mating traits traded off, sexual selection was inhibited from occurring in the mating pool in some cases. This tended to occur when few males had the resource trait, particularly if many (but not all) males had the mating trait. Specifically, if the sum of the proportions of resource and mating traits was less than or equal to one, selection could not act upon the mating trait (Figure 4b).

### 3.3 Overall selection associated with pre‐mating pool and mating pool dynamics

**FIGURE 5:**
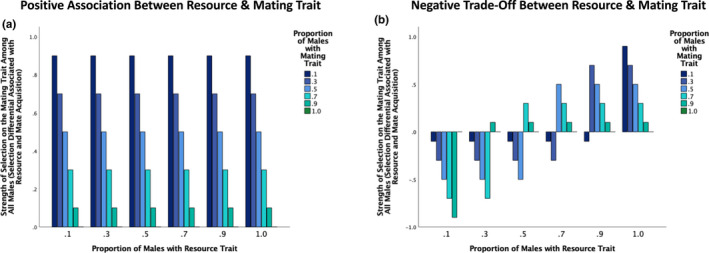
The strength of selection on a male mate‐acquisition trait in relation to both pre‐mating pool resource acquisition and mating pool dynamics. The selection differential associated with a mating trait across six levels of resource‐trait abundance and six levels of mating‐trait abundance (proportion of males with the resource trait = 0.1, 0.3, 0.5, 0.7, 0.9, or 1, proportion of males with the mating trait = 0.1, 0.3, 0.5, 0.7, 0.9, or 1) when there is (a) a positive association and (b) a negative trade‐off between the resource and mating traits. To focus on the overall or combined selection that was associated with pre‐mating pool resource acquisition and with mate acquisition within the mating pool, all adult males were included in the selection differential calculations.

To explore the overall selection on the mating trait that was associated with both pre‐mating pool resource acquisition and direct mate acquisition, we quantified the selection differential on the mating trait including all adult males (i.e., those with and without a resource). This measure of selection provides a look at the overall strength of selection on the mating trait in relation to the two competitive steps (resource acquisition and direct mate acquisition) in our model. When there was a positive association between the resource and mating traits, the overall strength of selection on the mating trait increased as the proportion of males with the mating trait decreased, and there was no effect of the resource‐trait abundance on selection on the mating trait (Figure 5a).

When the resource and mating traits traded off, the overall selection on the mating trait was most likely to be positive when a large proportion of males had the resource trait and was most likely to be negative when a small proportion of males had the resource trait (Figure 5b). The effect of male mating‐trait abundance on overall selection on the mating trait depended on the proportion of males with the resource trait (Figure 5b). When all males had the resource trait, overall selection on the mating trait increased as the proportion of males with the mating trait decreased. In contrast, when 90%, 70%, 50%, and 30% of males had the resource trait, overall selection on the mating trait was positive and greatest when 30%, 50%, 70%, and 90% of males had the mating trait, respectively (Figure 5b). When 90%, 70%, 50%, and 30% of males had the resource trait, the mating trait was selected against when 10%, 10–30%, 10–50%, 10–70% of males had the mating trait, respectively (Figure 5b). When 10% of males had the resource trait, the mating trait was selected against when 10–90% of males had the mating trait. These results suggest that when many males acquire a resource and enter the mating pool, there can be selection for a mate acquisition trait even when there is a trade‐off between resource and mating traits. In contrast, when few males secure a resource and enter the mating pool, we would expect overall selection to act against a mate acquisition trait if there is a trade‐off between resource and mating traits. In general, a trade‐off between resource‐ and mate‐acquisition traits can strongly affect the conditions that lead to positive selection for a mating trait, and such a trade‐off can in some cases prevent selection for a mate acquisition trait (Figure 5b).

## 4 DISCUSSION

Previous work has illustrated that resource acquisition can have strong effects on sexual selection (e.g., Ahnesjö et al., 2001; Almada et al., 1995; Borg et al., 2002; Borgia et al., 1987; Clutton‐Brock et al., 2006; Emlen & Oring, 1977; Forsgren et al., 1996; García‐Berro et al., 2019; Gwynne & Simmons, 1990; Hasegawa et al., 2012; Klug et al., 2010; Kvarnemo, 1997; Lindström, 1988; Lindström, 2001; Mück et al., 2013; Pärssinen et al., 2019; Shuker & Kvarnemo, 2021; Tudor et al., 2018; Vitousek, 2009; Wacker & Amundsen, 2014; Wong et al., 2018). However, in general, we lack a clear conceptual understanding of when and how resource acquisition is expected to impact sexual selection. In the present study, we used a simple model to generate *a priori* expectations of the effect of resource acquisition on sexual selection. The results of our model suggest that pre‐mating pool resource acquisition and mate acquisition within the mating pool can act independently or interact to affect selection on phenotypic traits (Table 2). Furthermore, the effects of resource and mate acquisition on sexual selection will depend on whether resource‐ and mate‐acquisition traits (1) are positively associated, which would be expected if some males are of higher quality than others, or (2) trade‐off due to costs of trait investment (Table 2). Such effects of resource and mate acquisition on selection will also depend on the competitive episode considered (Table 2).

**TABLE 2:** Predicted effects of resource acquisition and mate acquisition on selection on male traits

Scenario	Predicted effect on selection
Positive association between resource‐acquisition and mate‐acquisition traits.	Selection in relation to resource acquisition (pre‐mating pool): The strength of selection on a resource‐acquisition trait will increase when a smaller proportion of males have the resource trait. Selection in relation to direct mate acquisition (mating pool): On average, when only mating pool dynamics are considered and sexual selection acts on a mating trait, sexual selection on a mate‐acquisition trait will increase when a small proportion of males have the mating trait and when a large proportion of males have the resource trait. When a very small proportion of males have the resource trait, sexual selection on a mating trait will be absent, particularly if a large proportion of males have the mating trait. Selection in relation to resource and direct mate acquisition (pre‐mating pool and mating pool): Overall selection on a mating trait will increase as the proportion of males with the mating trait decreases and will be unaffected by the proportion of males with the resource trait.
Negative trade‐off between resource‐acquisition and mate‐acquisition traits.	Selection in relation to resource acquisition (pre‐mating pool): The strength of selection on a resource‐acquisition trait will increase when a smaller proportion of males have the resource trait. Selection in relation to direct mate acquisition (mating pool): On average, when only mating pool dynamics are considered, sexual selection on a mate‐acquisition trait will be greatest when all males have the resource trait and a small proportion of males have the mating trait. When there is variation in the male trait, sexual selection will on average increase when few males have the resource and mating traits, but sexual selection will depend on the specific combination of male resource‐ and mating‐trait abundances. When a small proportion of males have the resource trait, sexual selection on a mating trait will be absent, particularly if many (but not all) males have the mating trait. Selection in relation to resource and direct mate acquisition (pre‐mating pool and mating pool): Pre‐mating pool and mating pool dynamics can interact to influence overall selection on a mate‐acquisition trait. When a large proportion of males acquire a resource, overall selection on the mating trait will typically be positive and increase as fewer males have the mating trait. When a small proportion of males acquire a resource, overall selection on the mating trait will typically be negative, and under such conditions, selection against the mating trait will be strongest when a large proportion of males have the mating trait.

When focusing only on mating pool dynamics, sexual selection on the mating trait will be affected by both the proportion of males who have the resource trait and the mating trait. On average, when there is a positive association between the resource and mating traits, sexual selection associated with direct mate acquisition will increase as the proportion of males with the mating trait decreases and as the proportion of males with the resource trait increases. This pattern occurs because when many males acquire resources but few have the advantageous mating trait, there are many males in the mating pool but only a few males can monopolize all female mates. This, in turn, creates strong nonrandom variation in mating success with respect to the mating trait within the mating pool. In some cases, resource acquisition can inhibit sexual selection in the mating pool. When few males have the resource trait and there is a positive association between the resource and mating traits, sexual selection in relation to mate acquisition will be absent, particularly if a large proportion of males have the mating trait. In such cases, few males acquire a resource and enter the mating pool, but those males all have the mating trait. This prevents sexual selection in relation to direct mate acquisition (Figure 4a), and all selection associated with mating occurs in relation to resource acquisition (Figure 3a). When considering the effect of resource and direct mate acquisition on the overall strength of selection on a male mating trait, selection will be greatest when few males have the mating trait across all levels of male resource‐trait abundance when there is a positive association between the resource and mating traits. Empirically, when some males are simply of higher quality than others, we would expect: (1) strong natural and/or sexual selection for a resource‐acquisition trait when there is a resource that is essential for mating (Figure 3a); (2) strong sexual selection within the mating pool if few males have the mating trait, particularly if many males acquire a resource (Figure 4a); and (3) strong overall selection for an advantageous mating trait if few males have the mating trait across all levels of resource‐trait abundances (Figure 5a). These results are consistent with some empirical research. For example, sand goby males engaged in more courtship when nests were abundant, which would be expected if sexual selection within the mating pool became stronger when more males secured resources and were therefore in the mating pool (Forsgren et al., 1996). In the freshwater snail *Physa acuta*, reduced food was associated with reduced potential for sexual selection (Janicke et al., 2015).

When resource and mating traits trade‐off, sexual selection associated with direct mate acquisition is expected to be greatest when all males have the resource trait and few males have the mating trait. When there is variation in resource‐trait abundance, the strength of sexual selection will increase when few males have the resource and mating traits, on average, but the strength of sexual selection on the mating trait will depend on the specific combination of resource‐trait abundance and mating‐trait abundance considered. For example, when the resource and mating traits trade off and a small proportion of males have the resource trait, sexual selection is unlikely to occur within the mating pool, particularly if few males have the mating trait. When few males acquire a resource and the resource and mating trait trade off, few males enter the mating pool and none of those males have the mating trait. This prevents sexual selection from occurring with respect to direct mate acquisition (Figure 4b), and all selection will occur with respect to resource acquisition (Figure 3b). When considering the overall selection on a mating trait, there will be (1) relatively strong selection favoring the mating trait when many males have the resource trait, particularly if few males have the mating trait, and (2) relatively strong selection against the mating trait when few males can acquire a resource if the resource and mating traits trade off (Figure 5b). Empirically, when resource and mating traits trade off and few males can obtain a resource, we would expect little to no selection for a mating trait. Indeed, when few males can acquire a resource, which would be expected when competition for resources is strong, selection for a resource acquisition trait will inhibit selection for a mate‐acquisition trait. High levels of competition for resources that are required for mating might explain why sexual selection is absent within the mating pool in many species. Indeed, many species lack obvious traits associated with female choice or intersexual selection. Our finding that resources can influence sexual selection on a mating trait is also generally consistent with the finding that sex roles can be heavily influenced by resource availability in insects (Gwynne & Simmons, 1990; Simmons & Bailey, 1990).

In general, the above results provide a set of testable predictions of the effects of resource acquisition and direct mate acquisition on sexual selection (Table 2). Successful mating often involves multiple competitive steps (Ahnesjö et al., 2001), and each step in the mating process can influence the strength of selection on a mate acquisition trait. This is true regardless of whether resource acquisition is influenced by natural selection, sexual selection, or both natural and sexual selection. In the future, it will be important for empirical studies to explore the relative contribution of natural and sexual selection to traits that are associated with the acquisition of resources essential for mating, as well as the relative contribution of natural and sexual selection in shaping the overall or combined selection on mate‐acquisition traits.

Importantly, in the current study, we did not consider post‐copulatory sexual selection. It will be particularly interesting for research to examine how resource acquisition and mating pool dynamics can interact to influence subsequent selection on post‐copulatory mating traits. It will also be interesting for theoretical work to explore how the interaction between resource and mate acquisition can influence sex roles across trade‐off scenarios. Finally, the current model did not consider variation in adult population size. If the underlying trait distributions and mate sampling remain the same across resource‐ and mating‐trait abundances in our model, changes in adult density would not be expected to affect the qualitative patterns. That is, our qualitative patterns are expected to be robust to changes in adult density all else equal. However, previous research suggests that sexual selection can be density dependent (e.g., if harassment increases at greater densities (Kokko & Rankin, 2006)), suggesting that, in some cases, mate sampling might depend on density. It would be worthwhile for future theoretical work to expand on our simple models to consider density‐dependent mate acquisition, as well is variation in adult sex ratio and continuous traits.

## AUTHOR CONTRIBUTIONS

**Hope Klug:** Conceptualization (equal); data curation (supporting); formal analysis (supporting); funding acquisition (lead); investigation (lead); methodology (lead); project administration (lead); writing – original draft (lead). **Chelsea Langley:** Data curation (equal); formal analysis (equal); writing – review and editing (supporting). **Elijah Reyes:** Formal analysis (supporting); investigation (supporting); writing – review and editing (supporting).

## CONFLICT OF INTEREST

The authors have no conflicts of interest.

## Supporting information


Appendix S1

## Data Availability

All data have been deposited in Dryad: https://doi.org/10.5061/dryad.k98sf7m88.
